# Sequential Administration of Carbon Nanotubes and Near-Infrared Radiation for the Treatment of Gliomas

**DOI:** 10.3389/fonc.2014.00180

**Published:** 2014-07-15

**Authors:** Tiago Santos, Xin Fang, Meng-Tse Chen, Weijun Wang, Raquel Ferreira, Niyati Jhaveri, Martin Gundersen, Chongwu Zhou, Paul Pagnini, Florence M. Hofman, Thomas C. Chen

**Affiliations:** ^1^Department of Pathology, Keck School of Medicine, University of Southern California, Los Angeles, CA, USA; ^2^University of Coimbra, Coimbra, Portugal; ^3^Department of Electrical Engineering, Viterbi School of Engineering, University of Southern California, Los Angeles, CA, USA; ^4^Department of Chemical Engineering and Materials Science, Viterbi School of Engineering, University of Southern California, Los Angeles, CA, USA; ^5^Department of Neurological Surgery, Keck School of Medicine, University of Southern California, Los Angeles, CA, USA

**Keywords:** carbon nanotubes, near-infrared radiation, hyperthermia, glioma, glioblastoma multiforme

## Abstract

The objective was to use carbon nanotubes (CNT) coupled with near-infrared radiation (NIR) to induce hyperthermia as a novel non-ionizing radiation treatment for primary brain tumors, glioblastoma multiforme (GBM). In this study, we report the therapeutic potential of hyperthermia-induced thermal ablation using the sequential administration of carbon nanotubes (CNT) and NIR. *In vitro* studies were performed using glioma tumor cell lines (U251, U87, LN229, T98G). Glioma cells were incubated with CNTs for 24 h followed by exposure to NIR for 10 min. Glioma cells preferentially internalized CNTs, which upon NIR exposure, generated heat, causing necrotic cell death. There were minimal effects to normal cells, which correlate to their minimal uptake of CNTs. Furthermore, this protocol caused cell death to glioma cancer stem cells, and drug-resistant as well as drug-sensitive glioma cells. This sequential hyperthermia therapy was effective *in vivo* in the rodent tumor model resulting in tumor shrinkage and no recurrence after only one treatment. In conclusion, this sequence of selective CNT administration followed by NIR activation provides a new approach to the treatment of glioma, particularly drug-resistant gliomas.

## Introduction

Glioblastoma multiforme (GBM), World Health Organization (WHO) grade IV, is the most aggressive type of primary brain tumor (1). Median survival is only 12–15 months and 5-year survival is <5% regardless of treatment. Standard of care consists of surgery, followed by radiation and chemotherapy, usually temozolomide (TMZ). These therapies have significant side effects and routinely result in the recurrence of drug-resistant tumors (2, 3). Unfortunately, upon recurrence, patients have very limited treatment options. Chemotherapy with bevacizumab is often used as second line therapy (4). Further, ionizing radiation is generally not recommended, although radiosurgery boost has been used in some selected situations (5). Photothermal treatment is an alternative therapy, which induces cytotoxicity to drug-sensitive and -resistant tumor cells (6). This therapy involves the induction of hyperthermia, defined as temperatures above 40°C. This therapeutic intervention causes irreparable cell damage to tumor cells, while causing some damage to normal cells (7, 8). The main problem with hyperthermia is the difficulty in specifically targeting cell populations for destruction. Our approach to specificity is the use of carbon nanotubes (CNT) to regulate the levels of hyperthermia. CNTs possess particular electrical, optical, and thermal properties generated by the arrangement of the carbon atoms in a three-dimensional cylindrical nanostructure (9). These structures have strong optical absorptions in the near-infrared radiation (NIR) range (700–1400 nm) and generate heat through the release of vibrational energy. This property can be utilized for the induction of hyperthermia (10). Photothermal therapy for cancer has been a topic of recent investigation because this treatment strategy results in few side effects, limited invasiveness, and enhanced sensitivity of tumor cells to hyperthermia (8, 11). Wavelengths in the NIR range have a great advantage for *in vivo* applications because they cover the tissue-transparency window of the light spectrum (12). Furthermore, the low absorbance of NIR by water and biological tissues provides a favorable platform to irradiate CNTs. Hence, the combination of CNTs and NIR appears to be a promising therapy for glioblastoma treatment.

In this study, we demonstrate that the combination of CNTs and NIR is an effective photothermal therapy that selectively affects both drug-sensitive and -resistant glioma cells and tumor initiating glioma cancer stem cells (GSC), while sparing normal cells. Furthermore, these studies demonstrate that this therapy is effective *in vivo* for drug-resistant tumors without significant pathology to neighboring normal control tissues.

## Materials and Methods

### Cell culture and treatments

U251 TMZ-sensitive cells, U251 TMZ-resistant, U87 glioma cells, U87 TMZ-resistant, LN229 glioma cells, LN229 TMZ-resistant, and T98G glioma cells were cultured in 10% fetal calf serum (FCS; Omega Scientific Inc., Tarzana, CA, USA) in Dulbecco’s Modified Eagle’s Media (Corning, Santa Clara, CA, USA) supplemented with 100 U/mL penicillin and 0.1 mg/mL streptomycin. Human brain endothelial cells (BEC) and astrocytes were cultured in RPMI 1640 growth media (Mediatech Inc., Manassas, VA, USA) supplemented with 100 ng/mL EC growth supplement (Millipore, Temecula, CA, USA), 10 mmol/L *N*-2-hydroxyethylpiperazine-*N*-2-ethanesulfonic acid (HEPES) (Invitrogen, Carlsbad, CA, USA), 24 mmol/L sodium pyruvate (Invitrogen), 300 U heparin (Sigma-Aldrich, St. Louis, MI, USA), 1× minimum essential medium (MEM) vitamin solution (Invitrogen), 1× MEM non-essential amino acids (Mediatech Inc.), 1% penicillin/streptomycin (Invitrogen), and 10% FCS. Cancer stem cells were cultured in serum-free medium composed of Dulbecco’s modified Eagle medium [(DMEM)/F12 + GlutaMAX-I)] supplemented with 100 U/mL penicillin, 0.1 mg/mL streptomycin, 1% B27 supplement (Invitrogen), 20 ng/mL epidermal growth factor (EGF; PeproTech, Oak Park, CA, USA), and 20 ng/mL basic fibroblast growth factor-2 (FGF)-2 (Peprotech). Glioma cell lines were originally purchased from ATCC; TMZ-resistant cells were developed by serial passaging of tumor cells with increasing concentrations of TMZ. CNTs were purchased from Nanointegris (Nanointegris, Menlo Park, CA, USA). For cell treatments, cells were kept in culture in the presence of CNTs for 24 h prior to NIR exposure.

### Fluorescein-labeling of CNTs

Carbon nanotubes (Sigma-Aldrich) were shortened and carboxylated by heating in acid to yield highly functionalized nanotubes. Oxidized CNTs were reacted with fluorescein-5-thiosemicarbazide (FC) (1 mg/ml) and 1-ethyl-3-(3-dimethylaminopropyl)-carbodiimide (EDC) for 2 h at room temperature (RT). The mixture was filtered with a molecular weight cut-off of 100,000. The residue was washed with PBS several times and then collected and dispersed in PBS by sonication to make a suspension of CNT–FC of 0.25 mg/ml.

### Cell death assay

Cells were seeded at a density of 500 per well in 96-well plates; cells were then treated with increasing concentrations of CNTs (0.3–30 μg/mL) for 72 h. Supernatants and attached cells were collected separately and analyzed for necrosis and apoptosis, respectively, using commercially available ELISA kit per the manufacturers’ instructions (Cell Death Detection ELISA^PLUS^, Roche Applied Science, Indianapolis, IN, USA). For the propidium iodide (PI) incorporation assay, cells were incubated with PI for the remaining 20 min of the assay. Photos were taken using EVOS fl AMF-4306 AMG microscopes.

### Cell proliferation assay

Cells were seeded at a density of 1 × 10^4^ per well and grown for 24 h on 10 mm glass coverslips sitting in 24-well plates. CNTs (0.3, 1, and 3 μg/mL) were added as appropriate. Cells were treated with 5-bromo-2′-deoxyuridine (BrdU) (50 μM; Sigma-Aldrich) for the remaining 2 h of the assay. Cells were fixed with 4% paraformaldehyde and non-specific binding was prevented by incubating cells in 3% bovine serum albumin (BSA) and 0.1% Triton X-100 solution for 30 min at RT. Cells were incubated overnight at 4°C with mouse monoclonal anti-BrdU (1:50; Molecular Probes, Eugene, OR, USA), then washed with PBS, and incubated for 2 h at RT with Alexa Fluor 594 donkey anti-mouse (1:200; Molecular Probes) and Hoechst 33342 (2 μg/mL) (Sigma-Aldrich). All images were captured using EVOS fl AMF-4306 AMG microscopes.

### Tetrazolium dye (MTT) assay

Cells (2,000 or 500 cells per well for 72 h or 5 days MTT, respectively) were seeded in 96-well plates. CNTs were added for 24 h, and then exposed to NIR. MTT was performed for 72 h or 5 days after the sequential treatment. MTT assay was conducted according to the manufacturer’s protocol (EMD Chemical, Gibbstown, NJ, USA).

### Colony-forming assay

Glioma cells were seeded in 12-well slide chambers (IBIDI, Verona, WI, USA) at 200 cells per well. Subsequently, cells were treated with CNTs for 24 h and then exposed to NIR. At the end of 10 days, colonies were visualized by staining with 1% methylene blue in methanol for 1 h and quantified.

### *In vivo* studies

All animal protocols were approved by the Institutional Animal Care and Use Committee of the University of Southern California. Luciferase-labeled U251-TMZ-resistant glioma cells (5 × 10^5^ cells in 50 μL) were implanted subcutaneously. When tumors reached 12 ± 2 mm^3^, animals were imaged and randomly distributed into groups of four each, CNT treatment was performed by injecting intratumorally a total volume of 50 μL. One day after the CNT injection, a single NIR laser treatment (10 min at 6.75 W/cm^2^) was performed at the tumor site. Tumor sizes were measured every 2 days and mice were imaged weekly. For the imaging, mice were injected with 1 mg/kg Viviren™. *In vivo* Renilla Luciferase Substrate (Promega, Madison, WI, USA) administered intravenously and imaged using the IVIS 200 optical imaging system (Caliper Life Sciences, Hopkinton, MA, USA); images were analyzed using LIVING IMAGE software (Caliper Life Sciences).

### Statistical analysis

Statistical analysis was performed using GraphPad Prism 5.0 (GraphPad Software, San Diego, CA, USA). Statistical significance was considered relevant for *p* values <0.05 using one-way analysis of variance followed by Bonferroni or Dunnett *post hoc* test. Data are presented as mean ± standard error of the mean (SEM). Every experimental condition was tested in three sets of independent experiments unless stated otherwise, and performed in duplicates or triplicates.

## Results

### CNTs are not cytotoxic to glioma cells but decrease cell proliferation

**Figure 1:**
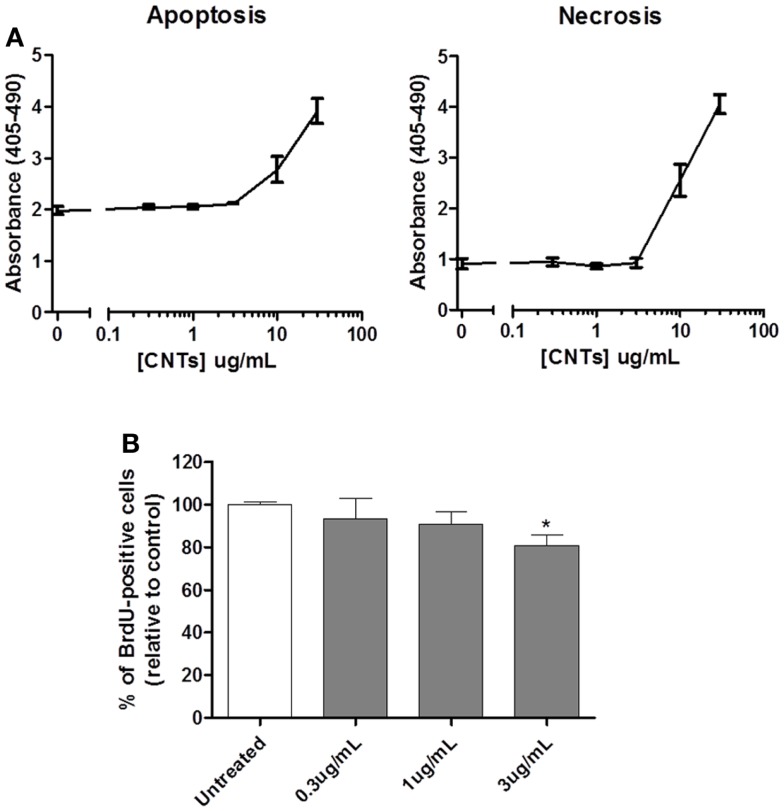
**Effects of CNTs on cytotoxicity and proliferation of glioblastoma cells**. **(A)** U251 glioma cells were treated with different doses of CNTs and evaluated after 72 h using the cell death ELISA. Doses of CNTs <3 μg/mL were not cytotoxic; apoptosis (left panel; *p* < 0.05; *n* = 3) and necrosis (right panel; *p* < 0.05; *n* = 3). **(B)** U251 glioma cells treated with CNTs (0.3–3 μg/mL) were evaluated for proliferation after 72 h using Brdu incorporation assay; 3 μg/mL significantly reduced cell proliferation (*n* = 3, *p < 0.05).

To determine whether exposure to CNTs alone induced cytotoxicity, U251 glioma cells were incubated with different concentrations of single-walled CNTs (0.3–30 μg/mL). Both apoptotic and necrotic cell death was evaluated 72 h after treatment using a cell death ELISA kit. The results (Figure 1A) show that CNTs did not induce either apoptosis or necrosis at concentrations equal to 3 μg/mL; higher doses of CNT demonstrated both apoptotic and necrotic cell death. Previous reports suggested that CNTs interact with filamentous actin (F-actin) monomers, causing a disruption of the cell cytoarchitecture and decreased proliferation (13). Therefore, the effects of CNTs on cell proliferation were also tested. Using the BrdU assay, the results demonstrated that CNTs, at 3 μg/mL, decreased cell proliferation by 20% as compared to untreated control cells (Figure 1B). Based on these data, 3 μg/mL was selected for further studies on the cytotoxic effects of the combination of CNTs and NIR.

### NIR-exposed CNTs induce hyperthermia

**Figure 2:**
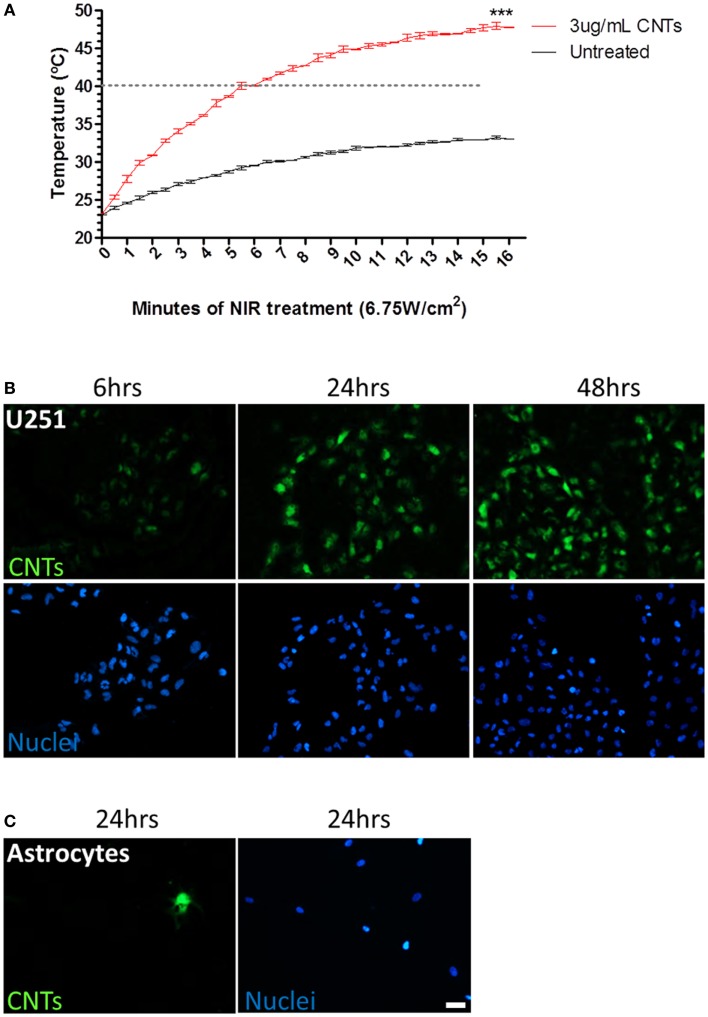
**Effects of sequential treatment of CNTs and NIR on hyperthermia and CNT internalization by tumor cells**. **(A)** U251 glioma cells treated with CNTs (3 μg/mL) and NIR (6.75 W/cm^2^) demonstrated an increase in temperature of the media compared to NIR alone (***p < 0.0001; *n* = 3). Dashed line represents hyperthermia threshold (40°C). **(B)** U251 glioma cells were incubated with fluorescein-labeled CNTs (green); maximum internalization was observed after 24 h. **(C)** Astrocytes were cultured with fluorescein-labeled CNTs for 24 h; few cells incorporated CNTs. Total numbers of cells were identified by Hoechst nuclear staining (blue). Scale bar is 20 μm.

We next investigated whether the highest non-cytotoxic dose of CNTs (3 μg/mL) was sufficient to significantly increase the temperature upon NIR laser irradiation (6.75 W/cm^2^). The value of 6.75 W/cm^2^ was selected since it was the highest power supported by the NIR laser used in this study, and therefore the most efficient in inducing hyperthermia. The hyperthermia threshold (40°C) was reached after 5 min of NIR irradiation in the presence of 3 μg/mL CNTs, while the untreated aqueous solution never reached the lethal 40°C threshold, even after 15 min of exposure to NIR. At 15 min, the temperature of the culture media alone increased from 23 to 33.02 ± 0.02°C; by contrast in the presence of CNTs (3 μg/mL), the media significantly increased from 23 to 47.76 ± 0.06°C (Figure 2A).

### CNTs are preferentially internalized by tumor cells

Since the internal concentration of CNTs may be critical to NIR susceptibility, the internalization kinetics were evaluated using fluorescently labeled CNTs. U251 glioma cells were treated with fluorescently labeled CNTs (3 μg/mL) and evaluated after 6, 24, and 48 h of treatment (Figure 2B). Optimal internalization was detected after 24 h; longer incubation time did not increase the intracellular signal significantly. Control normal human astrocytes were also tested for their ability to internalize CNTs. Unlike U251 cells, few astrocytes internalized CNTs (1 per 100 cells counted) (Figure 2C), indicating that CNTs are selectively internalized, with a preference for GBM cells as compared to normal astrocytes.

### Hyperthermia is toxic to glioma cells

**Figure 3:**
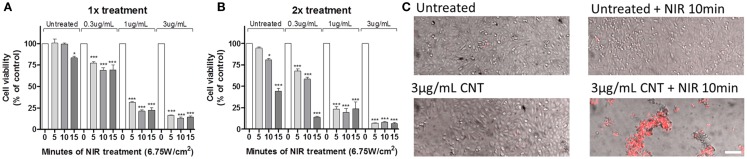
**The sequential treatment of CNTs and NIR induces lethal hyperthermia**. U251 glioma cells were treated with CNTs (0.3, 1, and 3 μg/mL) and NIR once **(A)** or twice **(B)** for 5, 10, or 15 min; after 72 h, the MTT cell viability assay showed that exposure to NIR (6.75W/cm^2^) reduced cell survival (*n* = 3, *p < 0.05, ***p < 0.001 relative to 0 min of NIR of each condition). **(C)** U251 glioma cells treated with CNTs (3 μg/mL) and NIR (10 min at 6.75 W/cm^2^) for 72 h exhibit necrotic cell death as shown by propidium iodide uptake (PI, red). Scale bar is 50 μm.

To evaluate the effects of hyperthermia on GBM, U251 cells were incubated with 0.3, 1, and 3 μg/mL of CNTs for 24 h, subsequently an NIR laser (6.75 W/cm^2^) was shone for a pulse of 5, 10, or 15 min (Figure 3A). Cell cultures were then evaluated after 72 h using the MTT assay. The results show that 10 min of NIR with 3 μg/mL produced the maximum decrease in cell viability (13.38 ± 0.83%). To determine whether a second exposure would enhance cell death, NIR treatment was repeated 24 h after the first exposure (Figure 3B). Except for 15 min, which was also toxic to untreated (no CNTs) cells, there was no significant difference between one or two exposures to NIR. Hyperthermia-induced cell death was dependent on both dose of CNTs and time of NIR exposure (Figures 3A,B).

All subsequent experiments presented in this study used the parameters of 24 h treatment of CNTs (3 μg/mL) followed by a single 10 min NIR exposure (6.75 W/cm^2^). This selection was based on the fact that these settings have an NIR exposure that is safe for untreated cells (99.69 ± 0.92% of control; Figure 3A), but induces a higher cell death when combined with CNTs (13.38 ± 0.83% of control; Figure 3A). Figure 3C depicts representative images of U251 glioma cells 72 h after exposure to different treatments; subsequently, cells were incubated with PI, which labels (red) necrotic dead cells. Only in the presence of the combination of CNTs and NIR did the number of PI-positive cells increase, with the majority of PI-negative cells exhibiting a rounded morphology with multiple detached cells, indicative of cell stress. Although these cells were not PI-positive, their metabolism was likely compromised.

**Figure 4:**
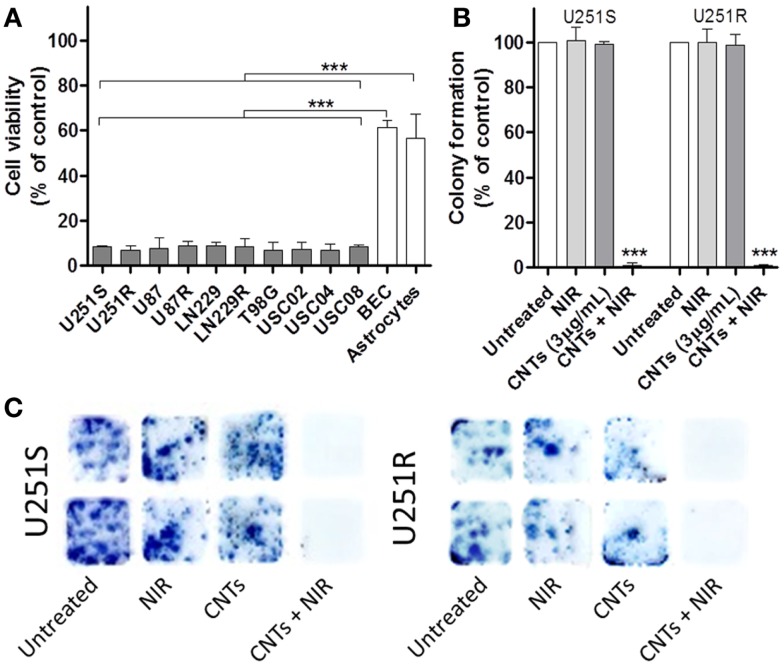
**Effects of CNTs and NIR on several different glioma cells compared to normal cells**. **(A)** GBM cell types (U251S, U251R, U87, U87R, LN229, LN229R, T98G), GBM cancer stem cells (USC02, USC04, USC08) and normal cells (human BEC and astrocytes) were treated with CNTs (3 μg/mL) and a single NIR treatment (10 min at 6.75 W/cm^2^). After 5 days, cell viability was evaluated using the MTT assay. Normal cells demonstrated greater viability compared to tumor cells (*n* = 3, ***p < 0.001). **(B)** U251 drug-sensitive and drug-resistant (U251R) cells were treated as described above. After 10 days, the numbers of colonies were significantly reduced in CNTs (3 μg/mL) plus NIR (10 min at 6.75 W/cm^2^) treated cells (*n* = 3, ***p < 0.001); **(C)** Representative images of the colonies are depicted.

We next tested the long-term effects of the combination of CNTs and NIR exposure on a variety of cells using the MTT assay for 7 days. Cell death was evaluated on the following cell cultures: U251 TMZ-sensitive cells (U251S), U251 TMZ-resistant (U251R), U87 glioma cells, U87 TMZ-resistant (U87R), LN229 glioma cells, LN229 TMZ-resistant (LN229R), T98G glioma cells, human BEC, human astrocytes, and primary GSC isolated from three different human specimens (USC02, USC04, and USC08) (Figure 4A). The results showed that photo-induced hyperthermia is effective in killing different glioma cell lines, including GSC (U251S: 8.34 ± 0.29%, U251R: 6.70 ± 1.18%, U87: 7.70 ± 2.77%, U87R: 8.70 ± 2.02%, LN229: 8.53 ± 1.13%, LN229R: 8.20 ± 2.15%, T98G: 6.86 ± 1.9%, USC02: 7.36 ± 3.13%, USC04: 6.86 ± 1.47%, and USC08: 8.20 ± 0.52%). Cell toxicity was achieved independently of TMZ-resistance status. By contrast, normal brain cells (BEC and astrocytes) exhibited no such cytotoxicity compared to the tumor cell populations (BEC: 61.49 ± 2.95%, astrocytes: 56.77 ± 10.57%). Thus, *in vitro* studies showed that CNTs-induced hyperthermia is less cytotoxic to normal healthy brain cells, as compared to tumor cells. Overall, tumor cell survival *in vitro* was decreased to levels below 10% throughout all glioma-derived cell types that were tested (Figure 4A). We also assessed the effects of this combination therapy on clonogenic survival using the colony-forming assay (CFA), a long-term viability assay (10 days) that measures the ability of tumor cells to survive, proliferate, and form colonies. CNTs (3 μg/mL) and NIR laser alone (6.75 W/cm^2^; 10 min) had no statistically significant effects on colony-forming ability (Figure 4B). However, the combination of CNTs and NIR exposure caused a reduction in viability to 0.926 ± 0.93% in U251S and to 0.762 ± 0.38% in U251R (*P* < 0.0001).

### The sequential administration of CNTs and NIR reduces tumor growth *in vivo*

**Figure 5:**
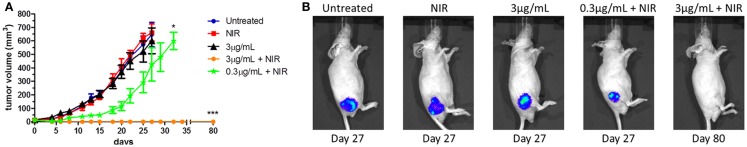
**The sequential, combination therapy with CNTs and NIR inhibits tumor growth *in vivo***. Athymic nude mice implanted with U251R luciferase-positive cells were either left untreated or treated with NIR (10 min at 6.75 W/cm^2^) alone, CNTs alone (3 μg/mL), CNTs (3 μg/mL) + NIR, and CNTs (0.3 μg/mL) + NIR; four animals/group. **(A)** Tumor size was measured every 2 days; day 0 accounts for the day mice were treated and followed up. Control groups (untreated, treated with CNTs alone, or treated with NIR alone) were statistically identical. Only treatment with CNTs (3 μg/mL) + NIR showed tumor remission (*n* = 4; *p < 0.05, ***p < 0.001 relative to untreated group). **(B)** Representative images of mice at day 27 exhibited tumors. By contrast, animals treated with CNTs (3 μg/mL) + NIR group did not show any tumor (*n* = 4) even at the termination of the experiment (day 80).

We next investigated the effects of CNT-induced hyperthermia in reducing tumor growth *in vivo*. Athymic nude mice were implanted with U251R renilla luciferase-positive cells into the hind flank. When tumors reached 12 ± 2 mm^3^, animals were distributed into five experimental groups: untreated, NIR alone (10 min, 6.75 W/cm^2^); CNTs alone (3 μg/mL, 50 μL injected intratumorally); CNTs (3 μg/mL, 50 μL injected intratumorally) + NIR; CNTs (0.3 μg/mL, 50 μL injected intratumorally) + NIR. Twenty-four hours after injection, tumors were exposed to NIR radiation (10 min, 6.75 W/cm^2^). Tumor growth was monitored and animals were euthanized when the tumor grew beyond 1.5 cm in diameter or the animals showed signs of stress or discomfort. The control groups of untreated, NIR alone-, CNTs alone-treated animals showed a similar tumor growth rate with no statistical differences (Figure 5A). By contrast, a significant dose-dependent inhibition of tumor growth was obtained in animals treated with CNTs plus NIR. The effectiveness of this therapy was dose-dependent and more efficient at the highest concentration of CNTs tested (3 μg/mL), where a total elimination of the tumor was observed. Furthermore, there were no signs of tumor recurrence up to 80 days after treatment (Figure 5B). Animals treated with a 10-fold lower concentration of CNTs (0.3 μg/mL) and NIR also exhibited a significant inhibition of tumor growth, however, not as dramatic as that observed with 3μg/mL CNTs in combination with NIR. These data demonstrate that sequential administration of CNT and NIR is effective *in vivo* and has clinical potential for patients with recurrent, drug-resistant gliomas.

## Discussion

The mechanism of toxicity induced by CNTs is controversial and may depend on different parameters such as nanotube type, size, shape, presence of impurities, and route of administration (14). The nanotubes used in our study were prepared by arc discharge and presented a 1.4 nm mean diameter and 1 μm mean length; these CNTs were non-toxic when used in concentrations up to 3 μg/mL. As previously reported by others, CNTs did significantly decrease cell proliferation, but only at 3 μg/mL. (13, 15).

Our data clearly show that CNTs, at non-cytotoxic concentrations, generate heat when exposed to NIR in a time-dependent fashion (Figure 2A). Notably, we detected a preferential uptake of CNTs by glioma cells versus their healthy counterparts, astrocytes (Figures 2B,C). GBM is a highly infiltrative tumor. Tumor cells migrate deeper into surrounding normal healthy brain. This characteristic makes the surgical resection a complicated task, leading either to an incomplete resection (and concomitant recurrence) or an excessive resection (damaging eloquent brain regions). Therefore, a therapy that could target specifically GBM cells would be beneficial for the complete eradication of the tumor. Although CNTs may seem an unlikely candidate, their specific uptake by GBM cells demonstrated here, justify their therapeutic use. Differences in CNT uptake may be explained by the mechanism of internalization chosen by these cells. There are reports demonstrating that CNTs enter cells through endocytosis (16, 17), specifically via tip recognition through receptor binding (18). These receptors may include scavenger receptors, lectin receptors, and integrin receptors. Interestingly, up-regulated integrin signaling is common for several invasive cancer types, including glioblastoma (19), thus supporting our observations.

Single-walled CNTs induce hyperthermia by generating strong optical absorptions in the NIR region while biological tissues are fairly transparent to these wavelengths. NIR and CNT-induced cell death was predominately necrotic (Figure 3C); hyperthermia is known to induce necrosis (7). Recently, graphene nanoparticles were shown to be more efficient in inducing lethal hyperthermia to U251 cells *in vitro* than CNTs (20). However, no uptake studies were performed on these cells; it would be of particular interest if the same preferential uptake observed by us was also observed with graphene nanoparticles. The efficacy of CNTs and NIR was reported for different types of cancer (6, 21–23). However, it is still unclear if this strategy can be used for glioblastoma. Wang and colleagues studied the efficacy of CNTs conjugated with CD133 antibody to target GBM cancer stem-like cells for photothermolysis (24). However, they did not compare the efficacy of their conjugated CNTs with unconjugated ones. The CNTs used by us were efficiently internalized by both GBM stem cells and tumor cells without the need of conjugation. Moreover, the *in vivo* effect reported by Wang et al. was obtained by pre-treating cells with CNTs before grafting them, whereas we performed a therapeutic approach. In our study, we only injected CNTs after confirming that the tumor was established. Furthermore, our treatment was equally efficient on both therapy-resistant cells and GSC, highlighting the clinical relevance of this therapy. We show here that GBM-derived cells were more sensitive to hyperthermia than normal BEC and astrocytes. These data are in accordance with previous studies, which showed that selective tumor killing is achieved at temperatures between 40 and 44°C, while most normal tissues remain undamaged at temperatures of up to 44°C for as long as 1 h (8).

As a proof of concept for clinical use, we performed *in vivo* experiments using human TMZ-resistant glioma cells in the xenograft subcutaneous rodent tumor model. Drug-resistant cells were used because this is the most challenging malignant population to treat. In this mouse model, CNTs were delivered intratumorally. The following day, the NIR laser exposure was initiated (day 0 in Figure 5A). Similarly to the *in vitro* data, a maximum uptake of CNTs was observed 24 h after administration (Supplementary Figure 1). A single 10 min hyperthermia treatment was sufficient for the observed dramatic reduction in tumor size (Figures 5A,B). Animals pre-treated with CNTs and exposed to NIR generated a small skin lesion, which disappeared within 24–48 h, leaving no visible mark on the skin. The depth of the lesion was related to the concentration of CNTs injected; this was a major consideration in determining the final dose of CNTs. All animals treated with this single sequential combination therapy at 3 μg/mL exhibited tumor shrinkage and ultimately no detectable tumor as confirmed by bioluminescence imaging. Animals treated with a 10-fold lower CNT concentration (0.3 μg/mL) showed a significant tumor regression albeit not complete. These data are in accordance with our *in vitro* results and other publications where the effects of CNT and NIR treatment were concentration-dependent (6, 11, 25).

From a clinical standpoint, the use of hyperthermia in brain tumors has been called laser interstitial thermal energy. Two companies, Monteris Medical (Plymouth, MN, USA) and Biotex (Houston, TX, USA) have both developed sophisticated high energy lasers for hyperthermia treatment. The problem with both systems is that local energy is deposited to a focal area using an introduced probe into the brain. As a result, each treatment requires a new invasive procedure. The use of high energy lasers also raises the risk of injury to normal cells in a non-targeted fashion. We envision our CNT treatment, followed by NIR, to be selective, less invasive, and potentially repeatable. Specificity can be obtained by local introduction of CNTs into the tumor cavity after surgical resection. Non-invasive treatment can be obtained by using external NIR-directed stereotactically into the tumor bed. Lastly, repeatability is most likely possible, as long as CNTs remain in the tumor bed, and are taken up by existing tumor cells after the previously irradiated tumor cells die. In our anticipated clinical application, we propose to deliver the CNTs locally rather than perform a systemic delivery, thereby avoiding the challenges of the blood–brain barrier (BBB). However, studies performed by Yang and colleagues revealed that single-walled CNTs are able to cross the BBB likely due to their nanostructure and particularly their nanoneedle-like shape. In their study, CNTs were found to be internalized by brain cells by transmission electron microscopy (26).

There are a number of potential problems with this therapy that still need to be resolved. First, an *in vivo* orthotopic glioma model must be explored to demonstrate the superiority of this approach compared to “standard” hyperthermia. Second, the specificity of CNTs for tumor cells versus normal cells over the long-term is not known. CNTs left in the tumor cavity may migrate out to the adjacent normal brain and induce undesirable by-stander effects to the normal brain not envisioned at the time of treatment. However, the advantage of leaving CNTs in the tumor bed enables NIR application on a repeated basis. Lastly, the effect of the skull in NIR is not certain. Other studies have shown that NIR is capable of penetrating the skull (27). This skull penetration by NIR would be the ideal scenario. One alternative would be to leave the bone flap off, and place it intra-abdominally.

In conclusion, the sequential combined therapy using CNTs and then NIR is a powerful cancer therapy for the elimination of malignant cells with minimal effects on normal tissues. Furthermore, all GBM cells tested internalized CNTs and were sensitive to hyperthermia, independent of their drug resistance status. Our approach opens promising perspectives for the treatment of therapy-resistant gliomas and possibly other cancer types.

## Conflict of Interest Statement

The authors declare that the research was conducted in the absence of any commercial or financial relationships that could be construed as a potential conflict of interest.

## Supplementary Material

The Supplementary Material for this article can be found online at http://www.frontiersin.org/Journal/10.3389/fonc.2014.00180/abstract
