# Supplementary material for: Sequential Administration of Carbon Nanotubes and Near-Infrared Radiation for the Treatment of Gliomas
Source: Front Oncol. 2014 Jul 15;4:180. doi: 10.3389/fonc.2014.00180 (PMC4097104; doi:10.3389/fonc.2014.00180)
Supplement: Supplementary file 1 [file Presentation1.PDF]

Supplementary Material

Sequential Administration of Carbon Nanotubes and Near Infrared Radiation for the Treatment of Gliomas

Tiago Santos[1,2], Xin Fang[3], Meng-tse Chen[3], Weijun Wang[4], Raquel Ferreira[4], Niyati Jhaveri[1], Martin Gundersen[3], Chongwu Zhou[3], Paul Pagnini[4], Florence M Hofman[1,4]*, Thomas C Chen[1,4]*

[1]Department of Pathology, Keck School of Medicine, University of Southern California, Los Angeles, CA,USA;
[2]University of Coimbra, Coimbra, Portugal;
[3]Department of Electrical Engineering and Department of Chemical Engineering and Materials Science, Viterbi School of Engineering, University of Southern California, Los Angeles, CA, USA;
[4]Department of Neurological Surgery, Keck School of Medicine, University of Southern California, Los Angeles, CA, USA;

*** Correspondence:** Thomas C. Chen, Departments of Neurosurgery and Pathology, University of Southern California, 2011 Zonal Avenue, Los Angeles, CA 90033. Phone: 323-442-3918; Fax: 323-442-3049; Email: tcchen@usc.edu. **And** Florence M. Hofman, Department of Pathology, University of Southern California, 2011 Zonal Avenue, Los Angeles, CA 90033. Phone: 323-442-1153; Fax: 323-442-3049; Email: hofman@usc.edu.

Supplementary Data

1. Supplementary Figures and Tables

1.1. Suplementary Figure

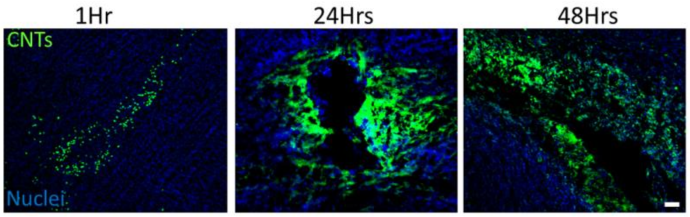

Supplementary Figure 1. Distribution of CNTs in the *in vivo* subcutaneous U251-resistant xenograft. Subcutaneous tumors were injected with 50µL of fluorescein-labeled CNTs (3µg/mL). Cellular uptake was followed along the needle track. Maximum internalization was observed after 24 hours. Scale bar is 100µm.
